# Coronary Artery Anomalies in Tetralogy of Fallot Patients Undergoing CT Angiography at a Tertiary Care Hospital

**DOI:** 10.7759/cureus.10723

**Published:** 2020-09-29

**Authors:** Tariq Ashraf, Faiza Farooq, Afaque Syed Muhammad, Parveen Akhtar, Mubashir Khan, Amin M Khuwaja, Muhammad N Khan, Musa Karim

**Affiliations:** 1 Cardiology, National Institute of Cardiovascular Diseases, Karachi, PAK; 2 Adult Cardiology, National Institute of Cardiovascular Diseases, Karachi, PAK; 3 Paediatric Cardiology, National Institute of Cardiovascular Diseases, Karachi, PAK; 4 Anaesthesia and Intensive Care, National Institute of Cardiovascular Diseases, Karachi, PAK; 5 Interventional Cardiology, National Institute of Cardiovascular Diseases, Karachi, PAK; 6 Statistics, National Institute of Cardiovascular Diseases, Karachi, PAK

**Keywords:** tetralogy of fallot, coronary artery anomalies, ct angiography, tertiary care hospital

## Abstract

Background

The aim of this study was to determine the frequency of coronary artery anomalies (CAAs) in Tetralogy of Fallot (TOF) patients undergoing computed tomography (CT)-angiography in a tertiary care hospital.

Methodology

In this observational study, we included consecutive TOF patients undergoing CT-angiography without prior history of cardiac surgery or congenital heart disease. CAAs were defined based on either origin or course of the artery.

Results

Out of 441 TOF patients, the prevalence of CCAs was 3.6% (16), of which 13 were below 18 years of age. Anomalous left main artery was observed in six (1.4%) patients, followed by left anterior descending artery and right coronary artery, observed in four (0.9%) patients each, and two (0.5%) patients had a single coronary artery originating from the left coronary cusp with an interarterial course.

Conclusions

CAAs were observed in a significant number (3.6%) of TOF patients. A CT-angiographic assessment before surgical correction would help identify the exact anatomy for better surgical planning to minimize complications.

## Introduction

Coronary artery anomalies (CAAs) are a complex group of congenital disorders that are highly variable in terms of clinical presentation and pathophysiological mechanisms and are not rare in congenital heart disease [1,2]. Tetralogy of Fallot (TOF) is the most common cyanotic congenital heart disease, accounting for 3.5 to 9% [3]. CAAs have been reported in 2 to 14% of TOF patients in coronary angiography, surgical and autopsy series [4]. In Pakistan, the true incidence of congenital heart disease is unknown due to limited access to medical care and limited resources to undertake intense population studies [5]. The reported incidence assessed by conventional angiogram is 5.6 to 8.4% [6]. Certain coronary anomalies are difficult to diagnose with TOF due to the complex anomalous origin and distribution of the coronary arteries [7]. These anomalies can add to morbidity and mortality during surgical repair [6]. The coronary anomalies of greatest concern are the origin of the left anterior descending coronary artery (LAD) from the right coronary artery (RCA) and certain variations of single coronary artery branching [2]. In order to improve surgical planning for better outcomes and to minimize the complications in tetralogy repair, effective imaging modalities are required to provide a thorough preoperative anatomic description of the associated intracardiac and extracardiac anomalies in TOF [4,7]. The identification of coronary anomalies can be documented by various techniques including autopsy, echocardiography, diagnostic coronary angiography, multislice coronary computed tomography (CT) angiography, or cardiac magnetic resonance imaging [8]. Of these, cardiac CT has particularly gained interest apart from echocardiography and MRI. Before the widespread use of coronary CT angiography, invasive catheter angiography was the standard for detecting CAAs [8]. Catheter angiography has obvious disadvantages, including its invasive nature and a small but finite risk of complications [8,9]. Coronary CT angiography is widely available and has proven to be an accurate and sensitive non-invasive alternative [9,10]. It provides excellent spatial resolution, fast acquisition time, no or little sedation, and an alternative to MRI in patients with pacemakers and implantable cardioverter defibrillators (ICDs) [9,10]. In the past, studies were conducted to assess CAAs in congenital heart diseases [11-14]. The current study assessed the incidence of coronary artery anomalies in patients with Tetralogy of Fallot by cardiac CT-angiography before total surgical correction in a cardiac tertiary care center.

## Materials and methods

This was an observational hospital-based study conducted at the National Institute of Cardiovascular Diseases (NICVD), Karachi. As per study inclusion criteria, 441 patients with Tetralogy of Fallot (TOF) who underwent cardiac CT-angiography were reviewed from the period of February 2012 to July 2017 with follow-up of two years. Exclusion criteria included patients who had undergone previous cardiac surgery and known forms of congenital heart diseases.

Study oversight

This study was approved by the Institutional ethics review committee (ERC) and conducted in compliance with Good Clinical Practice guidelines. The investigators agreed to maintain the confidentiality of the data. All the authors assure the completeness and accuracy of the data and data analyses.

CT angiography

All cardiac CT examinations were performed and interpreted by a consultant cardiologist with more than eight years of working experience. In all these patients CT examinations had been completed on a 64 slice dual-source scanner (SOMATOM Definition; Siemens, Germany) with the following scan parameters; 0.6 mm collimation, 80 kVp, and 30-80 mA with ECG dose modulation to minimize radiation exposure (typical dose of 5-8 mSv).

The scan coverage was from the brachiocephalic vessel to the upper abdomen. The contrast (ultravist 370) was injected through a peripheral intravenous catheter (18-20 gauge) at 4 ml/sec and with retrospective ECG gating via power injection. This was followed by a saline bolus. However each cardiac CT protocol varies slightly based on patient weight, associated lesions, prior surgical repair, and clinical questions asked at the time of examination. Scans were re-constructed with a slice thickness of 0.75-1.0 mm and a slice overlap of 0.6 mm.

Tetralogy of Fallot (TOF)

Patients presenting with clinical suspicion of TOF such as cyanosis, clubbing, recurrent chest infections, and heart murmur underwent echocardiography and TOF was confirmed with echocardiographic findings of collective occurrence of four of the related heart defects, which included right ventricular (RV) outflow tract obstruction (RVOTO; infundibular stenosis), ventricular septal defect (VSD), overriding of aorta, and RV hypertrophy.

Coronary artery anomalies (CAA)

Coronary artery anomalies were defined based on either origin or course of the main coronary arteries such as left main (LM), left anterior descending coronary artery (LAD), left circumflex artery (LCX), and right coronary artery (RCA). The origin or course were considered normal as per the definition described elsewhere [15].

Anomalous origin of coronary artery

The coronary arteries can have variant anomalous origins and courses such as anomalous locations of coronary ostium within the proper coronary ostium but either high or low to ST junction or at commissural level, or there can be anomalous location of the coronary ostium outside the normal sinuses which can be either right posterior aortic sinus (RCC), ascending aorta, LV, RV, pulmonary artery, or from any other major coronary artery. Lastly there can be anomalous origin of coronary ositum from the opposite facing coronary sinus, RCA arising from left coronary cusp (LCC) with anomalous course, LAD arising from the RCC with anomalous course, LCX arising from RCC with anomalous course, and LM arising from RCC with anomalous course [1,2].

Anomalous course of coronary artery

**Figure 1:**
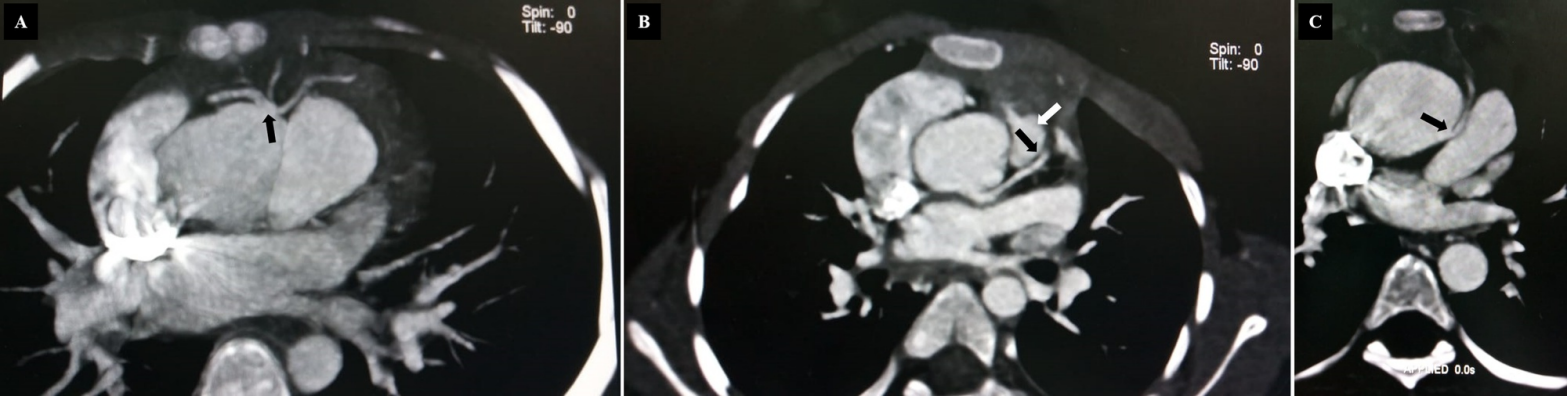
A) anomalous origin LAD from RCA, B) LAD passes close to pulmonic valve, and C) RCA originates from LCC with interarterial course. LAD=left anterior descending coronary artery, RCA=right coronary artery, LCC=left coronary cusp

The anomalous coronary arteries originating from the improper sinus can take any of these courses: retrocardiac (posterior atrioventricular groove), retroaortic, preaortic (intratrial), intraseptal (supracristal), prepulmonary (precardiac), or posteroanterior interventricular groove [1,2]. The coronary artery anomalies in patients with Tetralogy of Fallot by cardiac CT-angiography are presented in Figure 1.

Statistical analysis

Statistical Package for the Social Sciences (SPSS®) v21 (IBM Corp, Armonk, NY, USA) was used for the analysis of baseline characteristics. The frequency and percentages were calculated for categorical variables. The chi-square test was performed to evaluate the effect of age on study outcome. A two-sided p-value of less than 0.05 was taken as criteria for significance.

## Results

Baseline characteristics

In this study, we reviewed cardiac CTs of 441 consecutive TOF patients, of which 257 (58.1%) were male and the mean age was 11.66 ± 9.14 years. The majority (80%) of the patients were under 18 years of age and the remaining 20% were up to 45 years. Demographic characteristics of the TOF patients are presented in Table 1.

**Table 1:** Demographic characteristics of patients with Tetralogy of Fallot (TOF)

Characteristics	Total
N	441
Gender	
Male	256 (58%)
Female	185 (42%)
Age distribution	
Range [maximum - minimum]	1 month to 45 years
Mean ± standard deviation	11.66 ± 9.15
0 to 18 years	354 (80.3%)
19 to 45 years	87 (19.7%)

Anomalous coronary arteries

The prevalence of anomalous coronary arteries in TOF was found to be 3.6% (16), of which 13 were observed in patients under 18 years and the remaining three cases were observed in patients 18 to 45 years. The incidence of anomalous coronary arteries by gender was 4.3% (11/256) vs. 2.7% (5/185) (p=0.337) for male and female patients respectively.

Anomalous left main

The anomalous origin in the left main artery (LCA) was observed in six (1.4%) patients, all between 0-18 years of age. Left main (LM) was found to be originated from right coronary cusp (RCC) in three patients, whereas for the other three patients, each had its origin from non-coronary cusp (NCC), pulmonary artery, and sinotubular junction, respectively. Four of these anomalous originated left main arteries followed the normal course whereas for the remaining two, one covered the course through a retroaeortic route and one retropulmonic.

Anomalous LAD

The anomalous LAD was found in four patients out of which two arose from RCC and one from right coronary artery (RCA) whereas one had normal origin, i.e. left coronary cusp (LCC), but an anomalous course. In two of these patients, LAD followed an interarterial course, whereas it followed preaortic and retropulmonic courses in one patient each.

Anomalous RCA

The right coronary artery was anomalous in four patients, out of which two originated from NCC and from LCC and LAD in one patient each. The course of anomalous RCA was interarterial in two patients and retroaortic for the remaining two patients.

Two of the TOF patients had other anomalies such as single coronary artery from LCC with interarterial course. The most common course of anomalous coronary arteries was interarterial, often referred as the malignant course, comprised 37.5% (six out of 16), whereas the other 37.5% (six out of 16) appeared to be benign, consisting of retroaeortic in 18.8% (three out of 16), retropulmonic in 12.5% (two out of 16), and preaortic 6.3% (one out of 16) while four anomalous originated coronary artery followed the normal course and accounted for the remaining 25%. The distribution of anomalous coronary arteries is presented in Table 2.

**Table 2:** Distribution of Anomalous Coronary Arteries by Age Groups *Significant at 5% **p-values are based on chi-square test

Characteristics	Overall	Age Groups		**P-value
		0 to 18 years	18 to 45 years	
N	441	354	87	-
Anomalous Coronary Arteries	16 (3.6%)	13 (3.7%)	3 (3.4%)	0.055
Left main (LM) coronary artery				
Anomalous LM	6 (1.4%)	6 (1.7%)	0 (0%)	0.036*
Origin of Left Main [Base = LM Anomalous]				
Non coronary cusp (NCC)	1 (16.7%)	1 (16.7%)	0 (0%)	-
Pulmonary artery	1 (16.7%)	1 (16.7%)	0 (0%)	
Right coronary cusp (RCC)	3 (50%)	3 (50%)	0 (0%)	
Sinotubular junction	1 (16.7%)	1 (16.7%)	0 (0%)	
Course of Left Main [Base = LM Anomalous]				
Normal	4 (66.7%)	4 (66.7%)	0 (0%)	-
Retro-aortic	1 (16.7%)	1 (16.7%)	0 (0%)	
Retropulmonic	1 (16.7%)	1 (16.7%)	0 (0%)	
Left anterior descending coronary artery (LAD)				
Anomalous LAD	4 (0.9%)	3 (0.8%)	1 (1.1%)	0.49
Origin of LAD [Base = LAD Anomalous]				
Left coronary cusp (LCC)	1 (25%)	1 (33.3%)	0 (0%)	0.135
Right coronary artery (RCA)	1 (25%)	0 (0%)	1 (100%)	
Right coronary cusp (RCC)	2 (50%)	2 (66.7%)	0 (0%)	
Course of LAD [Base = LAD Anomalous]				
Inter arterial	2 (50%)	1 (33.3%)	1 (100%)	0.513
Preaortic	1 (25%)	1 (33.3%)	0 (0%)	
Retropulmonic	1 (25%)	1 (33.3%)	0 (0%)	
Right coronary artery (RCA)				
Anomalous RCA	4 (0.9%)	2 (0.6%)	2 (2.3%)	0.743
Origin of RCA [Base = RCA Anomalous]				
LAD	1 (25%)	1 (50%)	0 (0%)	0.135
Left coronary cusp (LCC)	1 (25%)	1 (50%)	0 (0%)	
Non coronary cusp (NCC)	2 (50%)	0 (0%)	2 (100%)	
Course of RCA [Base = RCA Anomalous]				
Inter arterial	2 (50%)	2 (100%)	0 (0%)	0.046*
Retro-aortic	2 (50%)	0 (0%)	2 (100%)	
Miscellaneous Coronary Anomalies				
Single coronary artery from LCC with interarterial course	2 (0.5%)	2 (0.8%)	0 (0%)	0.228

The telephonic follow-up of the 16 TOF patients with CCAs was made after 18.56 ± 6.43 months and loss to follow-up rate was 43.8% (7). Loss to follow-up rate was higher due the fact that most of these patients belonged to the remote rural areas of Pakistan. Out of the remaining nine patients, three (33.3%) of the patients underwent surgical correction and two (22.2%) patients died. Of the patients who died, one patient, a boy aged nine months, underwent systemic-to-pulmonary artery (SP) shunt and cause of death was shunt blockage. For the other patient, a girl age eight months, no cardiac-related surgery was performed and she died of non-cardiac causes.

## Discussion

The incidence of coronary abnormalities, i.e. CAAs, varies according to the method of detection applied by the investigators. Overall, in angiographic, surgical and autopsy series, coronary artery abnormalities have been reported in 2 to 14% of patients with TOF. Different studies have shown a low incidence of CAAs (2 to 5%) during surgery because of difficulties in detecting the course and origin of vessels intraoperative as compared to autopsy findings that show a high incidence, i.e. 5 to 9%. Assessment of the true incidence of CAAs is by cardiac CT angiography that helps to define the origin and course of the arteries in TOF, which is difficult or even impossible by invasive coronary angiography [16,17].

American Heart Association and American College of Cardiology (ACC) guidelines recommend cardiac CT angiography as class I recommendation as a non-invasive method for diagnosis of CAAs [18]. Another non-invasive technique for defining CAAs is by magnetic resonance coronary angiography, which has the advantage over cardiac CT angiography of not using contrast agents or ionizing radiation [19]. The disadvantages over CT angiography are lengthy acquisition time, availability, and increased cost. CT angiography as compared to conventional invasive angiography gives a true outline of origin and course of CAAs.

The reported incidence of coronary anomalies in past angiographic studies was 7.0%. The present study showed the overall incidence of coronary artery abnormalities with TOF in our population was 3.6%, which corresponds to past studies data including Fellows et al. (84 patients, incidence 5%) [20] and Dabizzi et al. (265 patients, incidence 9%) [21]. This shows that coronary anomalies in TOF patients are present in a significant number and this indicates the importance of pre-operative coronary assessment by angiographic methods to manage the operative complications, including morbidity and mortality [20,21].

In our study, the most common coronary artery anomaly was LM (1.4%) followed by RCA and LAD (0.9%) each and 0.5% gained anomalous origin as single coronary artery from left coronary cusp with interarterial course. The left main coronary artery anomaly is a rare congenital heart anomaly related to myocardial infarction (MI) and occurs in approximately one per 300,000 live births and represents 0.5% of all congenital heart defects [22]. Various studies have documented the most common CAAs to be LAD and LCX as a separate origin and incidence of 0.41% followed by LCX arising from the RCA with an incidence of 0.37% [2].

The course of CAAs has major clinical importance. Retroaortic, prepulmonic, and septal (subpulmonic) course anomalies appear benign, whereas LCA represents most commonly with a malignant course and carries a high risk for sudden cardiac death (SCD) [23,24]. Less frequently associated with SCD is RCA from left coronary sinus is more prevalent (0.1%) [25]. In the present study, the most common course of anomalous coronary arteries was interarterial (37.5%), whereas retroaeortic was 18.8% and the rest were retropulmonic (12.5%) and preaortic (6.3%). Four anomalous originated coronary artery followed the normal course and accounted for the remaining 25%.

The existence of an anomalous course of coronary artery with TOF influences the choice of surgical strategy for complete repair. Inadvertent injury to an unrecognized coronary artery during repair is associated with poor outcomes [26]. The objective of surgery is adequate relief of RVOT obstruction with preservation of the coronary artery. Once the course of the anomalous coronary artery is delineated the surgical strategy is tailored according to the situation [27]. The immediate concerns are injury to the coronary artery resulting in myocardial ischemia, heart failure, or death [26] as well as RVOT obstruction because of inadequate relief limited by an anomalous coronary artery course [27]. In the long term, residual RVOT obstruction and re-intervention are the concerns. The available surgical options are to do a staged repair with SP shunt as palliation; this approach may give a chance for improvement in the patient's physical health and the subsequent possibility for the use of RV and PA conduit, however this may not affect the natural history of disease [28], a combination of trans-atrial and trans pulmonary approach which may give best results despite the possibility of high RV/LV pressure ratio [27,29], use of an RV to PA conduit thus avoiding the area where the anomalous coronary artery is and finally translocation of the main pulmonary coronary artery [27]. Given the impact and anomalous coronary artery has on the conduct of surgery for TOF as well as the short- and long-term outcomes, it is imperative to identify the course of coronary arteries during evaluation of a patient for surgery, and with the increasing availability of a CT coronary angiogram it is possible to outline the exact course of both coronary arteries and plan the surgery accordingly.

Lost to follow-up of patients were more in their clinical course regarding their outcomes. Due to low incidence of CAA, reported statistical associations and p-values can be over- or underestimated.

## Conclusions

The results of the present study demonstrate a 3.6% incidence of TOF-associated coronary anomalies in a routine cardiac clinic. The coronary artery anomalies constitute a diverse group of abnormalities, ranging from anatomic variants to those having hemodynamic consequences. The assessment by cardiac CT-scan before surgical correction to identify the exact anatomy and known variation helps to improve surgical planning for better outcomes and minimize the complications by significantly reducing the morbidity and mortality during surgery.
